# Structures of Human DPP7 Reveal the Molecular Basis of Specific Inhibition and the Architectural Diversity of Proline-Specific Peptidases

**DOI:** 10.1371/journal.pone.0043019

**Published:** 2012-08-29

**Authors:** Gustavo Arruda Bezerra, Elena Dobrovetsky, Aiping Dong, Almagul Seitova, Lissete Crombett, Lisa M. Shewchuk, Annie M. Hassell, Sharon M. Sweitzer, Thomas D. Sweitzer, Patrick J. McDevitt, Kyung O. Johanson, Karen M. Kennedy-Wilson, Doug Cossar, Alexey Bochkarev, Karl Gruber, Sirano Dhe-Paganon

**Affiliations:** 1 Institute of Molecular Biosciences, University of Graz, Graz, Austria; 2 Structural Genomics Consortium, University of Toronto, MaRS Centre, Toronto, Ontario, Canada; 3 Department of Physiology, University of Toronto, MaRS Centre, Toronto, Ontario, Canada; 4 GlaxoSmithKline, Computational and Structural Chemistry, North Carolina, United States of America; 5 GlaxoSmithKline, Biological Reagents and Assay Development, Collegeville, Pennsylvania, United States of America; University of Cambridge, United Kingdom

## Abstract

Proline-specific dipeptidyl peptidases (DPPs) are emerging targets for drug development. DPP4 inhibitors are approved in many countries, and other dipeptidyl peptidases are often referred to as DPP4 activity- and/or structure-homologues (DASH). Members of the DASH family have overlapping substrate specificities, and, even though they share low sequence identity, therapeutic or clinical cross-reactivity is a concern. Here, we report the structure of human DPP7 and its complex with a selective inhibitor Dab-Pip (L-2,4-diaminobutyryl-piperidinamide) and compare it with that of DPP4. Both enzymes share a common catalytic domain (α/β-hydrolase). The catalytic pocket is located in the interior of DPP7, deep inside the cleft between the two domains. Substrates might access the active site *via* a narrow tunnel. The DPP7 catalytic triad is completely conserved and comprises Ser162, Asp418 and His443 (corresponding to Ser630, Asp708 and His740 in DPP4), while other residues lining the catalytic pockets differ considerably. The “specificity domains” are structurally also completely different exhibiting a β-propeller fold in DPP4 compared to a rare, completely helical fold in DPP7. Comparing the structures of DPP7 and DPP4 allows the design of specific inhibitors and thus the development of less cross-reactive drugs. Furthermore, the reported DPP7 structures shed some light onto the evolutionary relationship of prolyl-specific peptidases through the analysis of the architectural organization of their domains.

## Introduction

Maturation of many biologically important peptides, including those with neuro/vasoactive and immuno-regulatory activities, requires removal of an N-terminal X-Pro (residues P2 and P1, correspondingly) dipeptide. The enzymes possessing this highly specific activity [1] are called proline-specific dipeptidyl peptidases (DPPs). At least some, if not all proteins in this family play important roles in the regulation of signaling by peptide hormones and are involved in metabolic processes associated with diabetes, oncology and hematology [2].

Archetypal of this family and the most extensively studied member, DPP4, is a clinically successful target for drug design. DPP4 inhibitors constitute a new generation of medicines for type 2 diabetes, with some of them approved in more than 40 countries [3]. Other dipeptidyl peptidases are often referred to as “DPP4 activity- and/or structure-homologues” (DASH); these comprise DPP4 (the founding member), DPP7 (also known as DPP-II and quiescent cell proline dipeptidase, QPP), DPP8, DPP9 and fibroblast activation protein-α (FAP) [4]. All DASH members belong to the serine protease superfamily.

**Figure 1:**
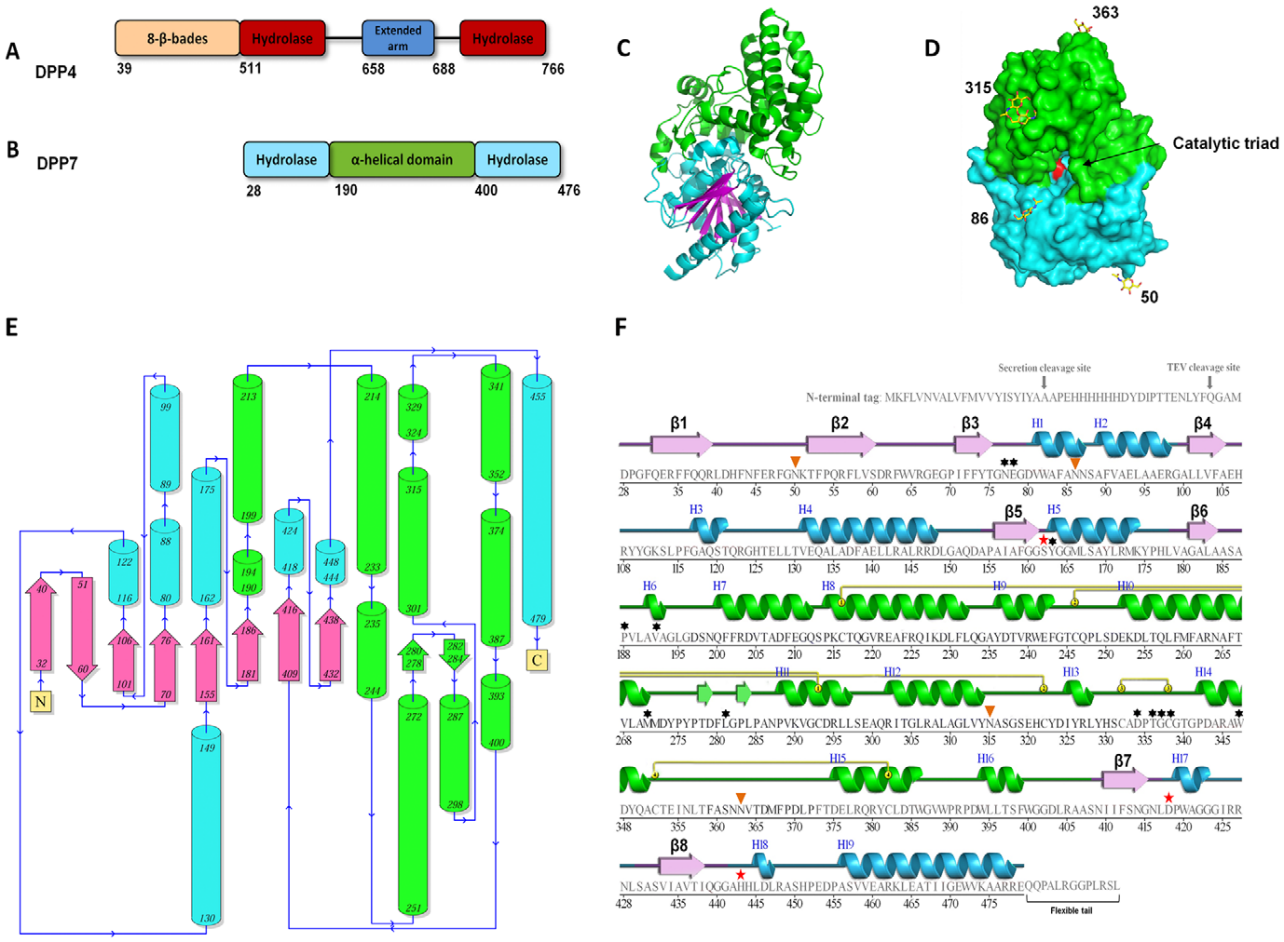
Structure of DPP7. (**A**) Schematic showing the DPP4 domain structure. The domains are represented as boxes and their borders are indicated. The propeller domain is in yellow, the hydrolase domain in dark red and the extended arm in blue. (**B**) Schematic showing the DPP7 domain structure aligned with that of DPP4. The hydrolase domain is in aquamarine and the big α-helical (SKS) domain in green. (**C**) Ribbon presentation of the DPP7 protomer structure. The domains are colored as in (B) with the β-strands characteristic for the hydrolase fold presented in magenta. (**D**) Surface representation of the DPP7 protomer. The domain is colored as in (B) and (C). The catalytic triad (Ser162, Asp418 and His443) is shown in red. The carbohydrates identified in the molecule are represented as sticks and colored ‘per atom’ (yellow, blue and red for C, N, and O, respectively). The corresponding amino acid numbers are shown in black. (**E**) Topology diagram evidencing how the new fold is positioned in relation to the catalytic fold. Color code is the same of Figure 1. (**F**) Expressed sequence information. Secondary structure of DPP7 was aligned to the amino acid sequence. Residues without secondary structure are not observed and presumed flexible. The color code is the same as in the previous figures. The catalytic triad (Ser162, Asp418 and His443) is indicated by a “red star”. The strands are represented by arrows and helices by bars. The glycosylated residues identified in the electron density map are marked with orange triangles and the suggested inhibitor interacting residues with asterisks. Disulfide bonds are indicated by yellow circles linked to the corresponding partners by yellow bars. Items **C** and **D** were prepared using the program PyMOL (http://www.pymol.org/).

DPP4 is a widely distributed glycoprotein observed in secreted and membrane-bound forms [5] and vast three-dimensional structural information is available for this enzyme [6], [7]. The functional form of DPP4 is a homodimer [8] with each protomer consisting of an N-terminal transmembrane anchor (membrane-bound form only) and two domains, an N-terminal eight-bladed β-propeller lobe and a C-terminal α/β-hydrolase domain (**Figure 1A**) [7]. The catalytic site is located in the cleft between the hydrolase and propeller lobes. The serine-protease active triad comprises Ser630, Asp708 and His740. Substrate specificity is mediated by residues contributed by both lobes.

The ubiquitously expressed DPP7 shares functional similarity with DPP4 [9], although they exhibit only low sequence similarity (11% identity and 26% similarity for the α/β-hydrolase domains only). DPP7 possesses a broad pH optimum, between 5.5 and 7.0, and is localized to intracellular vesicles [10], [11]. The predicted catalytic triad comprises Ser162, Asp418 and His443. DPP7 is the first reported protease that contains a leucine zipper motif through which the functional homodimer has been predicted to be formed [12]. Two N-glycosylation sites, Asn50 and Asn315, have been experimentally characterized and four more sites predicted by sequence analysis [13]. DPP7 is essential for maintaining vitality of lymphocytes and fibroblasts, and its inhibition results in apoptosis [14]. Its activity is also essential for preventing hyperinsulinemia and maintaining glucose homeostasis [15]. Physiological studies have shown that neuropeptides like casomorphin and bradykinin and their fragments are cleaved by brain DPP7 [16]. No natural DPP7 activators or inhibitors have as yet been reported, but many synthetic inhibitors of DPP7 are known, some of which were initially designed as inhibitors for DPP4 [17]. This cross-reactivity raises a concern, because inadvertent, concomitant inhibition of DPP7 may offset the desired effects [15].

Members of the DASH family have overlapping substrate specificities. Therefore, structural and biochemical analysis of other members should facilitate the development of specific, synthetic binders that can be used to elucidate the physiological roles of the DASH family members and/or to facilitate the structure-based drug design of pharmaceutically relevant inhibitors. Here, we report the structures of human DPP7 in its apo and inhibitor bound forms and compare them with structures of DPP4.

## Results and Discussion

### Overall structure of human DPP7

The structure of human DPP7 was determined from two crystal forms. Orthorhombic crystals (space group *P*2_1_2_1_2_1_) grown from selenomethionine labeled protein expressed in CHO-lec cells yielded diffraction data extending to a maximum resolution of 2.0 Å. These data were used to solve the phase problem by SeMet-MAD. The structure was then refined to a working R-factor of 20.8% (R_free_ 23.1%) with two chains in the asymmetric unit.

Another DPP7 construct without its N-terminal signal peptide (comprising of residues 28–492) was expressed as a secreted protein from insect cells, purified and crystallized in its ligand-free form (space group *P*2_1_) as well as in complex with the specific inhibitor L-2,4-diaminobutyryl-piperidinamide (Dab-pip). This structure was solved by molecular replacement (MR) using the 2.0 Å resolution structure as the template and refined to a working *R*-factor of 18.2% (*R*_free_ 22.2%) at 2.2 Å resolution. Co-crystallization with Dab-pip yielded monoclinic crystals isomorphous to those of the ligand-free protein. The structure of the inhibitor complex was refined at 2.45 Å resolution to a working *R*-factor of 22.1% (*R*_free_ 26.0%). Both monoclinic crystal forms contain four DPP7 chains in the asymmetric unit. Details of the structure determinations are provided in the Materials and Methods section as well as in **Table 1**.

**Table 1:** Data collection and refinement statistics.

	ligand-free DPP7 @ 2.0 Å	ligand-free DPP7 @ 2.2 Å	complex
**Data collection**			
Beamline	IMCA 17-ID	APS 19-ID	FR-E (Rigaku)
Wavelength (Å)	0.9798	0.9794	1.5418
Unit cell	*a* = 61.08 Å	*a* = 80.22 Å	*a* = 80.55 Å
	*b* = 96.44 Å	*b* = 130.22 Å	*b* = 130.50 Å
	*c* = 192.13 Å	*c* = 124.44 Å	*c* = 125.07 Å
		β = 102.4°	β = 102.3°
Space group	*P*2_1_2_1_2_1_	*P*2_1_	*P*2_1_
Matthews coefficient	2.56	3.05	3.09
Crystal solvent %	52	60	60
Resolution range (Å)*	500-2.0 (2.10-2.00)	50-2.2 (2.22-2.20)	20.0-2.45 (2.51-2.45)
Completeness (%)	98.87 (96.78)	100.0 (99.9)	99.5 (99.7)
Redundancy	6.2 (5.1)	9.8 (8.9)	3.3(3.1)
R_sym_	0.079 (0.475)	0.111 (0.688)	0.117 (0.793)
R_pim_	0.027 (0.294)	0.040 (0.213)	
R_rim_	0.072 (0.635)	0.129 (0.685)	
I/σ_I_	66.3 (3.2)	25.9 (3.3)	10.4 (2.1)
Unique reflections	72861	127772	92014
**Refinement**			
R/R_free_	0.206/0.236	0.180/0.222	0.221/0.260
r.m.s.-deviations			
bond length (Å)	0.009	0.007	0.010
bond angle (°)	1.142	0.85	1.07
Number of atoms			
protein	6981	14198	13833
ligand	-	-	52
water	411	946	248
Average B-factors (Å^2^)			
protein	32.67	32.90	41.62
ligand	-	-	36.29
water	35.15	38.03	34.79

* Values for the highest resolution shell are given in parentheses.

The structure of a DPP7 protomer is shown in **Figures 1B, 1C and 1D**. The protein has two domains: a classical catalytic α/β-hydrolase fold (residues 28–190 and 400–476; shown in aquamarine with β-strands in magenta) and a cap with an α-helical fold specific to the S28 protease family, which is connected to residues 190 and 400 of the hydrolase domain and referred to as SKS domain (shown in green in **Figures 1C, 1E and 1F**) in the human prolylcarboxipeptidase (PRCP, PDB code: 3N2Z) [18]. The predicted catalytic triad, Ser162, Asp418 and His443, is located in the interior of the protein, deep inside the cleft between the two domains (**Figure 1D**). Substrates appear to access the active site *via* a narrow tunnel. There were no significant structural differences between the protomers from any of the three crystal structures except for small changes in the active site (see below). The largest root-mean square deviation (RMSD) between any protomer was 0.4 Å for 408 superimposed Cα atoms (out of a total of 451 residues).

DPP7 and DPP4 (the archetype of the DASH family) share the catalytic α/β-hydrolase fold (**Figures 1A and 1B**). The environment of the active site, which is located in a cavity between catalytic and non-catalytic lobes, is different due to dissimilar architectural arrangements. In both DPP4 and DPP7, the scaffold of the catalytic pocket is formed by an eight-stranded β-sheet (shown in magenta in **Figures 1C, 1E, and 1F**). In DPP4, the catalytic domain comprises residues 511–658 and 688–766 (residues 28–190 and 400–476 in DPP7). The non-catalytic lobe of DPP4 is folded as an eight-bladed β-propeller domain assembled by the N-terminal residues 39 to 511 (**Figure 1A**). This propeller domain is not present in DPP7, which has the helical SKS domain [18] occupying the equivalent space instead. This domain originates from strand β6 of the α/β-hydrolase domain. The DPP4 equivalent for this feature is a small insert comprising residues 658 to 688 (**Figure 1A**) [7].

DPP7 has six potential N-glycosylation sites as deduced from the amino acid sequence (UniProt [19]). In the ligand-free structure crystallized in *P*2_1_, four of the predicted asparagine residues (50, 86, 315 and 363) were found to be glycosylated, displaying well-defined electron densities. In the orthorhombic structure only asparagines 315 and 363 were found to be glycosylated. No signs for glycosylation were observed in the electron density of the complex structure.

### Dimerization interface and its requirement for the enzymatic activity

It was previously shown that homodimerization is required for enzymatic activity of DPP7 and that residues from a leucine zipper motif are involved in oligomerization [12]. According to a PISA analysis [20] of all three crystal lattices, DPP7 may form stable dimers in solution. Because glycosylation appears to play a role in dimerization and because the monoclinic structure of the ligand-free enzyme had the largest content of glycans we focused our analysis on this particular structure.

**Figure 2:**
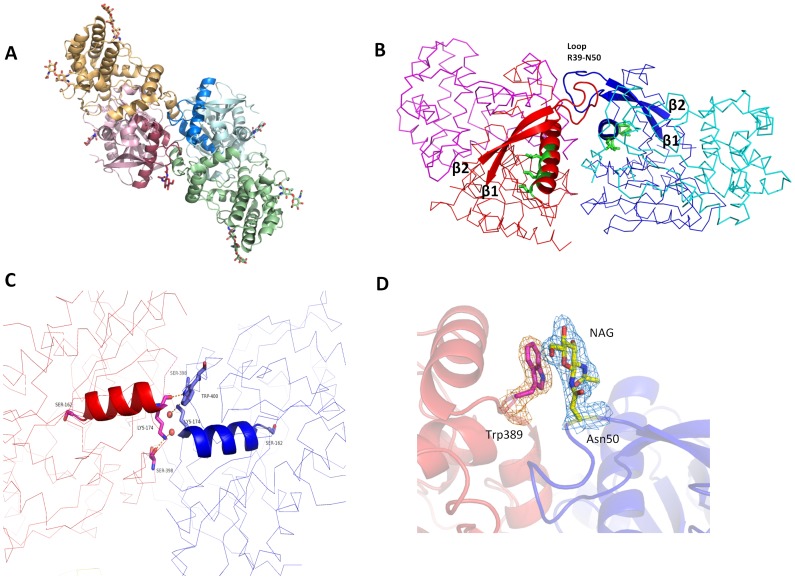
The DPP7 dimerization interface. (**A**) Cartoon representation of DPP7 overall structure highlighting the two protomers: helical domains are shown in orange and green, the α/β-hydrolase domains in red and blue. Carbohydrates are shown as sticks. (**B**) Dimerization mediated by the loop Arg39 – Asn50. Ribbon representation with two strands of the central β-sheet and the loop Arg39 – Asn50 represented as cartoon. The protomers are colored in blue and red, respectively. The supposed leucine zipper motifs are highlighted as cartoon and shown in green. (**C**) Dimerization mediated by helix α5. The catalytic Ser162 and residues participating in hydrogen bonds at the other of the helix are represented as sticks. Two water molecules are shown as red spheres. Helix α5 is represented as cartoon and the protomers are represented as ribbons in red and blue. (**D**) Stacking interaction between N-acetylglycosamine (NAG) linked to Asn50 and Trp389. A 2Fo-Fc electron density map of NAG and Trp389 is shown contoured at 1 σ. The figure was prepared using the program PyMOL (http://www.pymol.org/).

The two chains A and B (the biological assembly, **Figure 2A**) share an interface area of 2177 Å^2^ [20] with the major contribution arising from the loop Arg39-Asn50 responsible for 34% of the interface area (**Figure 2B**). The correct positioning of this particular loop (very likely acquired only through dimer formation) may be responsible for maintaining the integrity of the β-sheet in the α/β-hydrolase fold, since the loop connects strands β1 and β2. In addition, the catalytic serine (Ser162) is situated at the N-terminus of helix α5, amino acids of which contribute 280 Å^2^ (12%) to the interface area and form an extensive network of H-bonds with the second subunit (**Figure 2C**). Since several structure-function studies of serine proteases concluded that the precise geometry of the catalytic triad is essential for the activity [21], structural changes due to the disruption of the dimer interface may well explain the complete lack of enzymatic activity observed for monomeric DPP7 variants [12].

Correct glycosylation has also been shown to be required for DPP7 activity but not for its localization within the cell [22] indicating that it might play a role in dimerization. In our structures, the correct conformation of the loop Arg39-Asn50 appears to be attained due to the presence of an N-acetylglucosamine attached to Asn50 which stabilizes the loop conformation through stacking interactions with Trp389 (**Figure 2D**).

The leucine zipper motifs of each subunit, which were predicted to be crucial for dimer formation [12], indeed participate to some extent in the dimer interface but do not interact with each other as expected for leucine zipper motifs. Especially the leucines are pointing away from the second subunit (**Figure 2B**) rather than forming an interdigitated interaction. This arrangement completely differs from the classical description of dimer formation mediated by leucine zipper motifs [23].

### The catalytic domain – conservation of the catalytic triad

The catalytic domain is a representative of the ubiquitous α/β-hydrolase fold. It is formed by a β-sheet core of eight strands connected by α-helices, forming an α/β/α sandwich [24]. A Dali search [25] using only the catalytic domain of DPP7 indicated DPP4 as the third closest structural homologue (Z-score of 19.0), in spite of the low sequence identity of 11% (26% similarity) between the catalytic domains of both enzymes. A superposition extending over 204 Cα atoms resulted in an RMSD of 2.9 Å. The closest structural homolog to DPP7 is human prolylcarboxipeptidase also known as PRCP (PDB code: 3N2Z) [18] with a Z-score of 52.2, followed by the functionally unrelated feruloyl esterase from *Butyrivibrio proteoclasticus* (Z-score 19.8, PDB code: 2WTM) [26].

**Figure 3:**
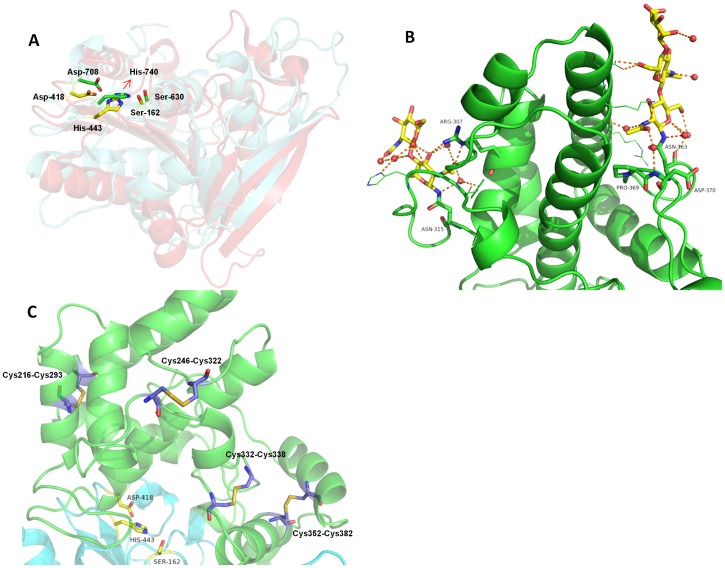
The catalytic domain and SKS domain. SKS domain shown in green cartoon and the catalytic domain shown in aquamarine cartoon. The catalytic triad is represented as yellow sticks. (**A**) Conservation of the catalytic triad evidenced by the structural superposition. The catalytic fold of DPP4 is shown as red cartoon. DPP7 catalytic residues (Ser-162, Asp-418 and His-443) are colored in yellow, while DPP4 catalytic residues (Asp-708, His-740 and Ser-630) in green. The corresponding amino acid numbers are shown in black. (**B**) The SKS domain is shown in green cartoon, while the glycans linked to Asn363 and Asn315 are shown as sticks. Both glycans seem to have a scaffold function by holding the loop Glu354-Asp375 to the helix α8 and the loop Asp324-Tyr314 to the helix α12, respectively. (**C**) Four disulfide bonds present in the SKS domain. Cys216-Cys293 and Cys246-Cys322 seem to have a structure stabilization role. Cys332-Cys338 is very likely involved in the activity regulation of the enzyme, while Cys352-Cys382 could play either regulatory and structural role. The figure was prepared using the program PyMOL (http://www.pymol.org/).

The catalytic domains of DPP4 and DPP7 were also superimposed using the ‘SSM Superimpose’ structural alignment function in COOT [27]. A visual inspection demonstrated the structural conservation of the catalytic triad (**Figure 3A**). Ser630, Asp708 and His740 in DPP4 are structurally equivalent to Ser162, Asp418, and His443 in DPP7. A list of solvent accessible surfaces of these residues are given in **Table S1**, hydrogen bonding distances between the Ser/His and His/Asp are listed in **Table S2**.

The catalytic triad residues of PRCP and DPP7 are also completely conserved, in this case including the neighboring residues His444 and Arg448 (corresponding to His456 and Arg460 in PRCP, respectively) (**Figure S3B**). It has been proposed for both enzymes that the arrangement of these residues in relation to the catalytic histidine (His455 in PRCP corresponding to His443 in DPP7) might play a role in the catalytic mechanism of both enzymes possible through modulation of the pKa value of the catalytic histidine [18].

### The DPP7 cap domain – a rare domain

The cap domain (green in **Figure 1** **and** **3**) is formed by 11 α-helices (α6 to α16) and two strands interconnected by loops, one of them being remarkably long (Glu354-Asp375). The glycan attached to the Asn363 appears to anchor this loop through extensive interactions involving helix α8 and the residues Pro369 and Asp370 and supported by a network of water molecules (**Figure 3B**). The glycan attached to Asn315 also seems to have a scaffold role, holding the loop Asp324-Tyr314 through interactions with the Arg307 in the helix α12, with water molecules also playing a role in the stabilization. The general fold of this domain has recently been described for the first time in the structure of human prolylcarboxipeptidase (PRCP) and has been denominated as an “SKS domain” [18].

Four disulfide bonds were identified in the cap domain all of which are proposed to play a role in stabilizing the structure (**Figure 3C**): Cys216-Cys293 holds the helices α8 and α11 together; Cys246-Cys322 stabilizes the loop Asn315- Asp324, which is also glycosylated; Cys332-Cys338 is situated in the loop insertion that defines the dipeptidyl aminopeptidase specificity of the enzyme (discussed below) and Cys352-Cys382 stabilizes the long loop mentioned above (residues 354 to 371). Based on experiments showing that DPP7 activity is affected by agents such as dithiothreitol (DTT), *p*-chloromercuribenzenesulfonic (PCMBS) as well as Hg^2+^ ions a free SH-group had previously been assumed to be involved in the catalytic mechanism of the enzyme [28], [29]. Our structural results, however, do not reveal such a free SH-group.

To date the S28 serine peptidase family is formed by only two enzymes (DPP7 and PRCP). They share 49% sequence identity and have recently been described as an “odd couple” of one enzyme cleaving at a Pro-X motif at the carboxy termini of proteins (PRCP) and another one cleaving X-Pro dipeptides off the amino termini of peptides (DPP7) [30].

We conducted a DALI search using only the cap domain of DPP7 (residues 190–400) to verify whether this structural feature is restricted to only this protein family. Beside DPP7 and PRCP themselves this analysis revealed the catalytic domain of a phosphodiesterase (PDB code 3SHZ) [31] as a potentially related structure but with a Z-score of only 3.1 and an RMSD of 3.9 Å. A Pfam search [32] using the sequence of the cap domain yielded “Peptidase S28 family” as the only significant match (E-value of 1.5e^−17^). Taken together, DALI and Pfam searches indicate that the SKS domain is indeed a rare fold possibly present only in the S28 serine peptidase family.

### Architectural diversity of the cap domain in Prolyl peptidases

**Figure 4:**
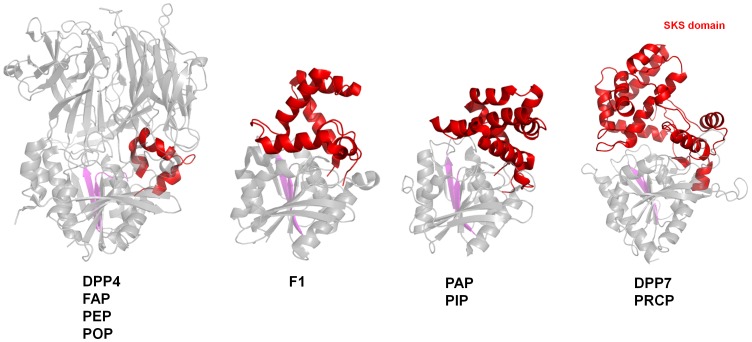
Representation of the evolution of the SKS domain. The α/β-hydrolase domain in all enzymes and the β-propeller domain in DPP4 are shown as grey cartoons. The cap domain in all enzymes are shown as red cartoon. DPP4, FAP, PEP and POP, share the same extended arm (red cartoon) which increases in complexity to become the cap domain in the F1 aminopeptidase. The degree of complexity is further increased in PAP and PIP, until it reaches its maximum convolution in the form of SKS domain in DPP7 and PRCP. For clarity purpose, only DPP4, F1, PAP and DPP7 are displayed in the Figure. β-strands 6 and 7 are shown in magenta. The figure was prepared using the program PyMOL (http://www.pymol.org/).

Regardless of the low sequence identity between DPP7 and DPP4 their 3D structures indicate that both enzymes are very likely related by divergent evolution. Similar domain organizations together with the close superimposition of the catalytic triad residues supports this hypothesis. The SKS cap domains in DPP7 and PRCP seem to have evolved through successive embodiments from a smaller feature as the extended arm seen in DPP4 (**Figure 1A** and **Figure 4**). The growth of this domain eventually led to the loss of the β-propeller as the cap domain. Prolyl peptidase fibroblast activation protein α (FAP) (PDB code: 1Z68, [33]), prolyl endopeptidase (PEP, PDB code 3IUJ) [34] and prolyl oligopeptidase (POP, PDB code 1QFM, [35]) share the same fold as DPP4 including the small extended arm.

Further evidence for the embodiment hypothesis comes from prolyl oligopeptidases displaying intermediate stages of the cap domain (**Figure 4**). The extended arm formed by 3 helices in DPP4, FAP, PEP and POP is augmented in complexity to 6 helices in the F1 aminopeptidase (PDB code: 1MTZ, [36]) and to further 8 helices in proline iminopeptidase (PIP, PDB code 1AZW, [37]) and prolyl aminopeptidase (PAP, PDB code: 1QTR, [38]). Finally, in DPP7 and PRCP, the cap domain reaches its maximum complexity with 11 helices, two strands and two N-glycosylation sites. In all these structures the cap domain is inserted between strands β6 and β7 of the α/β-hydrolase fold (**Figure 4**).

### The cavity system

**Figure 5:**
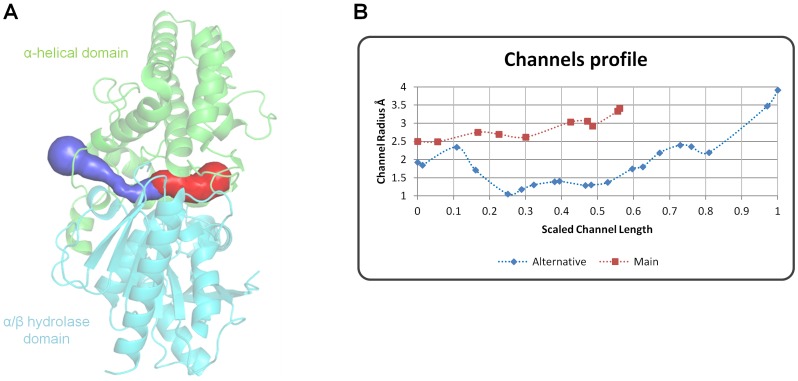
The channel system in DPP7. (**A**) The main channel is represented in red, and should be the route for the substrates to access the active site. It can be also visualized in Figure 1D. The products should be released either through the main channel or by an alternative channel (blue), identified by the Caver algorithm [39]. (**B**) Channels profile indicating the radius in Å *vs.* the scaled length, starting from the inhibitor position towards the protein surface. The figure was prepared using the program PyMOL (http://www.pymol.org/).

The cavity systems by which substrates access and products leave the active site differ significantly between DPP4 and DPP7. In DPP4, peptides pass either through a channel in the center of the propeller domain or through an opening between the hydrolase and the propeller domain [7] (**Figure S1**). In DPP7, substrates and products seemingly should pass through a single entrance between the hydrolase and cap domains (**Figure 1D** and **Figure 5A**). To evaluate the possibility of alternative channels in DPP7, we performed an analysis of its active site using the software Caver [39]. In addition to the main channel for the substrates (shown in red in **Figure 5A**), a putative exit path for the products (shown in blue in **Figure 5A**) was identified based on the channel profiles (**Figure 5B**). The residues lining these channels are indicated in **Table 2**. Although the radius of the alternative path initially decreases before becoming wider until it finally reaches the surface, the anatomy of channels might change as a consequence of substrate binding. For instance, aminopeptidase F1 (the 3^rd^ closest structural homolog of DPP7 according to DALI server, RMSD 2.9 Å, PDB code: 1MTZ) has two different openings: the always open main entrance and the alternative channel which is closed in the ligand-free protein. In this case, peptides are assumed to access the active site only *via* the main entrance, while the alternative channel which also connects the active site cavity with the protein surface was only observed in the ligand-bound form [36].

**Table 2:** Residues constituting the main and alternative channels in DPP7.

Channel	Residues
Main	Glu78, Asp80, Met271, Leu281, Thr336, Gly337, Cys338, Gly339, Thr340,Trp420
Alternative	Glu78, Pro188, Ala191, Val192, Gly194, Leu195, Gly196, Ser198, Asp334, Trp347, Asn356, Leu357, Thr358, Phe359, Asp375, Trp420

In summary, analysis of the structures suggests that DPP4 and DPP7 may have different mechanisms of peptide access and/or product release. While DPP4 has one entrance for substrates and a side opening for product release, DPP7 either has only one channel or might possess an opening mechanism triggered by substrate binding.

### Structure of DPP7 in complex with inhibitor

**Figure 6:**
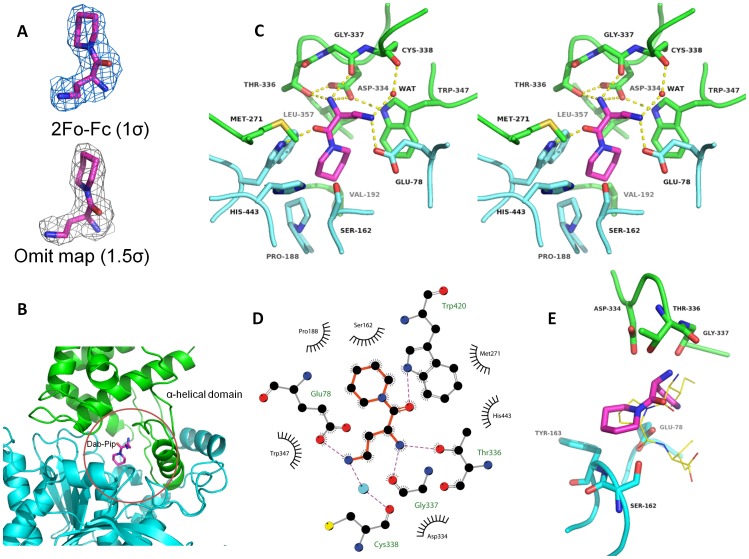
Inhibitor Dap-Pip in the DPP7 active site. (**A**) Omit and 2Fo-Fc electron density maps for the bound inhibitor are shown at 1 and 1.5σ, respectively. (**B**) General overview of the inhibitor in the binding pocket. The α/β–hydrolase domain is shown in aquamarine and the SKS–domain in green. The inhibitor is shown in magenta. (**C**) Stereo view of the residues involved in inhibitor binding. The coloring scheme is the same as above. Dashed yellow lines denote hydrogen bonding interactions (**D**) Representation of the interaction of the inhibitor with surrounding residues prepared using LigPlot+ [60]. (**E**) Superimposition of the DPP7 Dab-Pip complex with the structure of DPP4 complexed with Diprotin A [42]. The coloring scheme for DPP7 is the same as above. The Diprotin A is shown using thinner, yellow sticks. The figure was prepared using the program PyMOL (http://www.pymol.org/).

The DPP7 specific inhibitor L-2,4-diaminobutyryl-piperidinamide (Dab-Pip) binds noncovalently and has previously been identified by introducing a 2,4-diaminobutyric acid group at P2 and a piperidine moiety at P1 [40]. It has a half maximal inhibitory concentration (IC_50_) for DPP7 of 0.13 µM and for DPP4 of more than 1 mM [40]. In order to analyze the binding mode and determine the specific interactions occurring in the active site, we co-crystallized DPP7 with Dab-Pip and determined the structure of the complex at 2.45 Å resolution. Omit and 2Fo-Fc maps revealed clear electron density for this ligand (**Figure 6A**).

Both, the catalytic and the cap domain of DPP7 participate in inhibitor binding (**Figure 6B and C**). In addition to the catalytic triad – Ser162, Asp418, and His443 – the catalytic domain also provides Glu78, Pro188, Trp420 (carbons shown in blue), whereas the cap domain contributes Val192, Thr336, Gly337, Asp334, and Trp347 (green) (**Figure 6B and C**). While ten, highly networked water molecules fill the active site of the ligand-free form of the enzyme, water molecules W3, W4, W5, W6 and W7 were displaced by Dab-Pip (**Figure S2**). The precise location of three of these water molecules (W3, W5 and W6) coincides with three nitrogen atoms of the ligand, forming nearly identical interactions with the protein. Two water molecules (W1 and W2) are retained upon ligand binding, one of which (W1) acts as an interstitial water forming a hydrogen bond with one of the ammonium groups of the ligand and bridging it with the backbone carbonyl group of Cys338 (**Figure S2**).

The dipeptidyl aminopeptidase reaction specificity of DPP7 requires the necessarily unprotected and protonated N-terminus of the substrate peptide to be bound in an exact distance from the catalytic serine residue (Ser162), which allows the accommodation of two amino acids. The structure of the inhibitor complex indicates Thr336 and Asp334 to act as the N-anchor residues (**Figure 6B**). These amino acids are situated on an insertion (comprising residues Trp329 to Gly341) between the helices α13 and α14 of the cap domain.

Although the residues constituting the P1 pocket present a high degree of conservation between DPP7 and PRCP, the latter does not possess the insertion Trp329-Gly341 seen in DPP7, which contains the residues forming the P2 pocket. As a consequence, the active site of PRCP is larger, accounting for the different substrate and reaction specificities observed within this family of enzymes (**Figure S3A**).

The binding pockets of DPP4 and DPP7 are also significantly different arising mainly due to differences in the cap domains. Some functionally important residues lining the active site in DPP4 such as Tyr547 and Asn710 [41], which are provided by the propeller domain, are not conserved in DPP7. In spite of the totally different “selectivity” domains, DPP4 and DPP7 still retain functionally conserved residues participating in both the recognition of the N-terminus of the substrate (*i.e.* the S2 pocket) (**Figure 6B and D**) as well as the formation of the S1 pocket and its hydrophobic anatomy responsible for the selective acceptance of proline and its mimetics (**Figure 6C and D**). In the S2 pocket of DPP7, two anchor residues provided by the cap domain – Thr336 and Asp334 – are responsible for binding the N-terminus of the incoming peptide by hydrogen bonding and ion pair interactions (**Figures 6C and 6D**). An additional hydrogen bond is provided by the main chain carbonyl group of Gly337. In DPP4, Glu205 and Glu206 are the two main anchor residues, in this case provided by the β-propeller domain, and an extra hydrogen bond is formed through the hydroxyl group of Tyr662 [42].

In both DPP4 and DPP7 the S1 pocket (binding the P1 residue) is hydrophobic and small, accounting for the proline/alanine restriction at this position. In DPP7, this pocket is formed by Val192, Leu357, Pro188, Trp347 and Trp420 and accommodates the piperidyl-moiety of the inhibitor (**Figure 6C and D**). In addition, the carbonyl group of Dab-Pip forms a hydrogen bond with the indole NH-group of Trp420 (**Figure 6C and D**). In DPP4, the region structurally equivalent to Trp420 in DPP7 is occupied by Arg125, Asn710 and Glu205, facilitating the formation of 5 possible hydrogen bonds with a given ligand. In contrast, in DPP7, there is no other residue offering a possibility of extra hydrogen bonds with the oxygen. This is an evident region to be exploited in the design of more specific inhibitors.

**Figure 7:**
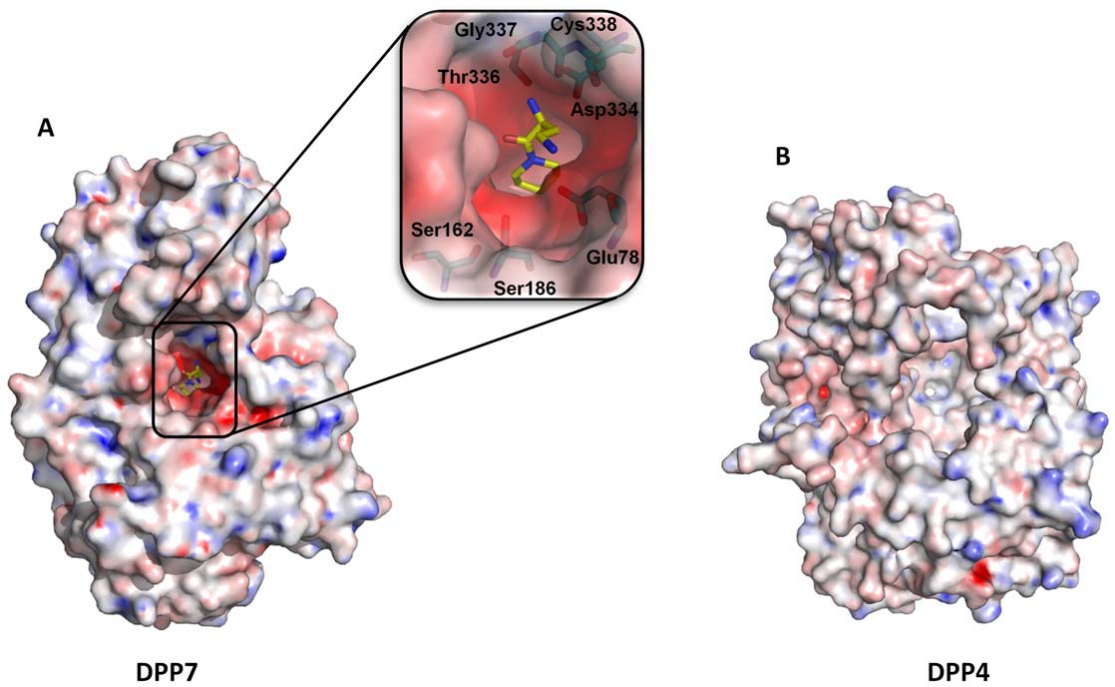
Poisson-Boltzmann electrostatic potential. (**A**) Surface representation of DPP7 showing the negatively charged substrate binding pocket. Inset: Zoom in the binding pocket depicting the amino acid residues that contributes to the negative charge. (**B**) Surface representation of the electrostatic potential of DPP4 evidencing the more neutral to positive charge in the binding pocket. The calculations were done with the chain A of DPP7 (PDB code 3JYH) and DPP4 (PDB code: 3EIO) using the software APBS [43]. The figure was prepared using the program PyMOL (http://www.pymol.org/).

The nitrogen at the 4 position of the 2,4-diaminobutyrate group (mimicking the side chain in the P2 position of a peptide substrate) forms a salt bridge with Glu78 (**Figure 6C and D**). It has been shown that DPP4 accepts a broader range of amino acids in this P2 position, whereas DPP7 is unable to accommodate negatively charged residues [11], a key aspect that can be explored for designing specific inhibitors. To address this, we performed Poisson-Boltzmann electrostatics calculations using the program APBS [43], which showed that DPP7 possesses a more negatively charged substrate binding pocket. This pocket is built up by the side chains of Glu78, Ser162, Ser186, Thr336 and Asp334, as well as the main chain of Gly337 and Cys338 (**Figure 7A**). Additionally, the S2 pocket of DPP7 is constricted due to the Trp329-Gly341 insertion and the helix α14 (Pro342-Glu354). In contrast, DPP4 presents a rather neutral and wide open substrate binding pocket, explaining the looser specificity of the enzyme (**Figure 7B**).

In order to further analyze the active site of DPP7 we superimposed the structure of the Dab-Pip complex with the structure of DPP4 complexed with Diprotin A (PDB: 1WCY) [42]. This compound (Ile-Pro-Ile) has been shown to inhibit both, DPP4 and DPP7 [44]. This superposition confirms the residues participating in the S2 and S1 pocket, as well as the residues involved in the N-anchor (**Figure 6E**). Additionally, it indicates the residues that constitute the “oxyanion hole” in DPP7. In DPP4, the mainchain amide of Tyr547 and the side chain of Tyr631 interact with the carbonyl group of Pro2 of Diprotin A and are thus assumed to also stabilize the tetrahedral intermediate [45]. In DPP7 the mainchain amide of Tyr163 and the sidechain of Glu78 are the most likely candidates for this role. Characteristically, DPP7 exhibits optimum activity at slightly acidic conditions (pH between 5.5 and 7.0) where Glu78 might be protonated.

### Conclusion

Our structural results provide a rationale for the difference in substrate specificity between DPP4 and DPP7, thus allowing the development of more specific and less cross-reactive drugs. Furthermore, the reported DPP7 structures shed some light onto the evolutionary relationships of prolyl-specific peptidases through the analysis of the architectural organization of their domains.

## Materials and Methods

### Construction of the secreted DPP7 expression plasmid

A construct encoding the fragment of human DPP7 containing amino acids 29–492 was amplified by PCR from DPP7 cDNA (MGC.AU64A9 BC011907) using primers with sequence 5′-CTGTATTTTCAGGGCGCCATGGATCCCGGCTTCCAGGAGCGC-3′ and 5′-CTCTAGTACTT CTCGACAAGCTTCATCAGAGGCTGAGTCTGGGCCC-3′. The amplified product was cloned into a pFHMSP-LIC-N donor plasmid which is a derivative of the pFastBac HT A vector (Invitrogen) for directing secreted protein expression in the Baculovirus Expression System in insect cells. The modified vector has the Honeybee melittin signal sequence upstream of a poly-His tag and a SacB gene stuffer sequence subcloned between Nco1and HindIII sites in the multiple cloning sequences of the pFastBac vector. The modified vector adds a 26 amino acid N-terminal fusion tag containing 6× His followed by a TEV cleavage site to the inserted coding sequence. The DPP7 29–492 fragment was inserted into the cloning/expression region of Nco1/HindIII linearized pFHMSP-LIC-N using Infusion (BD-Biosciences) enzyme mediated bidirectional recombination between complementary nucleotide DNA sequences at the ends of the insert (PCR product) and vector. Insertion of target sequence involved replacement of a SacB gene stuffer sequence, which provided for negative selection of the original plasmid on media containing 5% sucrose.

### Generation of recombinant Bacmid DNA and baculovirus

The recombinant donor vector pFHMSP-DPP7 was transformed into DH10Bac *E. coli* cells (Invitrogen) to generate recombinant viral DNA. Sf9 cells (Invitrogen) were transfected with Bacmid DNA using Cellfectin reagent (Invitrogen), and recombinant baculovirus particles were recovered. The recombinant virus preparation was sequentially amplified from P1 to P3 viral stocks.

### Expression and purification of recombinant DPP7 in insect cells

Sf9 cells grown in HyQ® SFX Insect Serum Free Medium to a density of 3×10^6^ cells/mL and with viability not less than 97% were infected with 20 mL of P3 viral stock for each 1 L of cell culture. Cell culture medium was collected after 4 days of incubation on a shaker at 100 RPM and 27°C when culture viability dropped to 25–45%. The culture was centrifuged at 14,000 *g* for 15 minutes, and the cell pellet discarded. The conditioned medium was adjusted to pH 7.5 at room temperature by adding 10× Buffer A (50 mM Tris pH 8.0, 0.15 M NaCl). Protease inhibitors were added to final concentrations of 1 mM phenylmethanesulfonyl fluoride (PMSF, Bioshop) and 2 mM benzamidine hydrochloride (Sigma). 1.6 L of conditioned medium was mixed with 20 mL pre-equilibrated NiNTA Superflow beads and stirred for 1 hour. The resin was transferred to a 100 mL gravity column, washed with 100 mL of Washing Buffer (50 mM Tris pH 8.0, 0.5 M NaCl, 2 mM imidazole) and the bound protein was eluted with 10 mL of Elution Buffer (50 mM Tris pH 8.0, 0.5 M NaCl, 250 mM imidazole). A second round of NiNTA batch absorption has been performed to increase protein yield. Protein eluted from the IMAC column was loaded onto a Superdex 75 16/60 gel filtration column (GE Healthcare) equilibrated with 50 mM Tris, 100 mM NaCl buffer pH 7.5. The chromatogram showed one major protein peak that consisted of DPP7 as confirmed by SDS-PAGE analysis. The protein was then TEV cleaved to remove the poly histidine tag. TEV was added in the ratio of 50∶1 DPP7∶TEV. The reaction was incubated at 4°C for ∼2 days. Cleavage was confirmed by SDS-PAGE analysis and the TEV and tag were removed by passing the sample through a 1 mL HisTrap FF crude column (GE Healthcare) which had been equilibrated with gel filtration buffer. Purified protein was concentrated to 3.5 mg/mL using concentrators with an appropriate molecular weight cut-off (Amicon Ultra-15 10,000 MWCO, Millipore). Average yield of DPP7 was about 2 mg/L.

### Expression and purification of DPP7 in CHO-lec cells

DPP7 (residues 1–492) with a C-terminal tev-Fc fusion was expressed in CHO-lec cells (specifically CHO Pro-Lec3.2.8.1.3B) using an Invitrogen pDEST vector. CHO-lec cells were obtained from Pamela E. Stanley (Department of Cell Biology, Albert Einstein College of Medicine, NY) [46].

The first 28 residues of DPP7 were cleaved, when the protein was secreted from the cells. The protein was collected on a ProSepA column. DPP7 was eluted from the column by Tev cleavage and further purified on a Superose 200 gel filtration column.

### Crystallization, data collection, structure solution, and model refinement of DPP7 from CHO-lec cells

Crystals of selenomethionine labeled DPP7 expressed in CHO-lec cells were grown by the hanging drop vapor diffusion method using VDX crystallization trays. The reservoirs contained 0.5 ml of the precipitating agent (0.1 M sodium acetate pH 4.6, 0–0.05 M zinc acetate, 4–10% Polyethylene Glycol (PEG) 3000–6000, 0–20% glycerol). A variety of drop ratios were employed using protein at 4–8 mg/ml. Rod-shaped crystals appeared within 2–5 days, but continued to grow over a period of approximately 2 weeks at 295 K.

For data collection, the crystals were cross-linked for 5 minutes at 295 K using 5 µl of 25% glutaraldehyde in a microbridge [47]. These crystals were frozen by immersion in liquid nitrogen using 0.1 M sodium acetate pH 4.6, 0.05 M zinc acetate, 5% PEG 3350, and 25% ethylene glycol as the cryoprotectant. Diffraction data was measured at Advanced Photon Source (APS) beamline IMCA 17-ID (Argonne National Laboratory). Data were processed with HKL2000 [48] and scaled in space group *P*2_1_2_1_2_1_ to 2.0 Å resolution. The spacegroup assignment was confirmed using the software POINTLESS [49]. The SeMet positions (12 per AU) were found with SHELXD [50] and refined with SHARP [51]. SOLVE/RESOLVE [52] and PHENIX [53] were used to apply NCS symmetry averaging and solvent flattening for the initial maps. The model was built with COOT [27] and refined with REFMAC [54] to an *R*_cryst_ of 20.6% (*R*_free_ 23.6%).

Clear electron density was observed for residues 28 through 477. The model was validated using MOLPROBITY [55] and deposited to the Protein Data Bank (PDB code: 4EBB). MOLPROBITY showed 95.6% of amino acid residues in the most favored region of the Ramachandran plot and only one outlier (Gly337), which acts as part of the N-anchor for the incoming peptide. The MOLPROBITY score was 96^th^ percentile (100^th^ percentile is the best among structures of comparable resolution). Data collection and processing details are listed in **Table 1**.

### Crystallization, data collection, structure solution, and model refinement of DPP7 from insect cells

Crystals of human recombinant DPP7 from insect cells were grown by vapor diffusion at 300 K using the sitting drops method. The crystal used for structure determination was grown in 2 M (NH_4_)_2_SO_4_, 0.2 M NaAc 0.1 M Hepes pH 7.5, 5% MPD. The inhibitor Dab-Pip (L-2,4-diaminobutyryl-piperidinamide) was synthesized as described in the literature [40]. For co-crystallization trials, DPP7 was mixed with 15 fold molar excess of Dap-Pip on ice for 30 minutes and crystallized under the same condition as without inhibitor.

Diffraction data were measured at Advanced Photon Source (APS) beamline 19ID (Argonne National Laboratory), and at an in-house X-ray source (FR-E+ superbright; Rigaku) respectively. The data were processed in space group *P*2_1_ to 2.2 Å resolution using HKL3000 [56] and to 2.45 Å resolution with HKL2000 [48] respectively. In both cases, the software POINTLESS [49] was used to confirm the space group assignment.

Molecular replacement was performed with the program MOLREP [57] using the coordinates of DPP7 crystallized in *P*2_1_2_1_2_1_ as the template. ARP/wARP [58] was used for automated model building. The model was further refined to an *R*_cryst_ of 18.2% (*R*_free_ 22.2%) by interactive rebuilding using COOT [27] and restrained refinement using REFMAC [54]. Clear electron density was observed for residues 28–479 in chains A and B, for 28–478 in chain C and 28–477 in chain D.

The final structure was validated with MOLPROBITY and deposited to the Protein Data Bank (PDB code: 3JYH). MOLPROBITY showed 95.8% of amino acid residues in the most favored region of the Ramachandran plot and displays 5 outliers. Except for Arg66, the density is clear for all outliers. In chain B, the outlier Leu417 is situated in the active site and is a neighbor of the catalytic Asp418. In the chain C, the outlier Ala442 is in the vicinity of the catalytic His443. The MOLPROBITY score was 98^th^ percentile (100^th^ percentile is the best among structures of comparable resolution).

The structure of DPP7 in complex with Dab-Pip was refined to an *R*_cryst_ of 22.1% (*R*_free_ 26.0%) by interactive rebuilding using COOT [27] and restrained refinement using BUSTER [59]. In chains A and B, clear electron density was observed for residues 28–479. In chains C and chain D, clear electron density was observed for residues 28–478 and 29–477, respectively. The final model was validated with MOLPROBITY and deposited to the Protein Data Bank (PDB code: 3N0T). MOLPROBITY showed 95.4% of amino acid residues are in the most favored region of the Ramachandran plot and displays only two outliers (Gly49 and Lys51). Both residues are located in a loop region. The MOLPROBITY score was 98^th^ percentile. Data collection and processing details are listed in **Table 1**.

## Supporting Information

Figure S1 **The channel system in DPP4.** The main channel is represented in red, and is assumed to be the route for the substrates to access the active site. The products are released through the alternative channel (blue), both channels are showed here as identified by the Caver algorithm [39]. DPP4 deposited under PDB code 1N1M was used for the channels calculation. (http://www.pymol.org/).
(TIF)

Figure S2 **Water molecules in the active site.** (A) Active site of ligand-free DPP7 with waters represented as red spheres. (**B**) Active site of DPP7 in complex with Dab-Pip with waters represented as red spheres and Dab-Pip as yellow sticks. Hydrogen bonds are shown as orange dashed lines. Water 1 acts as an interstitial water, bridging the interaction between the nitrogen at position 4 in the ligand with the carbonyl backbone of Cys338. The interacting residues are shown as lines. The figure was prepared using the program PyMOL (http://www.pymol.org/).
(TIF)

Figure S3 **Superposition of DPP7 and PRCP.** (**A**) Superposition of DPP7 (pink) and PRCP (blue), the insertion Trp329-Gly341 is shown as green sticks, while the catalytic Ser162 is depicted as a pink stick. (B) Zoom in the superposed active site of DPP7 (shown in pink) and PRCP (shown in blue). The corresponding amino acid numbers are shown in black.
(TIF)

Table S1 **Accessible surface area (in Å^2^) for the residues constituting the catalytic triad.**
(DOCX)

Table S2 **H-bonding distances between Ser-His and His-Asp of the catalytic triad.**
(DOCX)
