# Supplementary figures and images for: Structures of Human DPP7 Reveal the Molecular Basis of Specific Inhibition and the Architectural Diversity of Proline-Specific Peptidases
Source: PLoS One. 2012 Aug 29;7(8):e43019. doi: 10.1371/journal.pone.0043019 (PMC3430648; doi:10.1371/journal.pone.0043019)

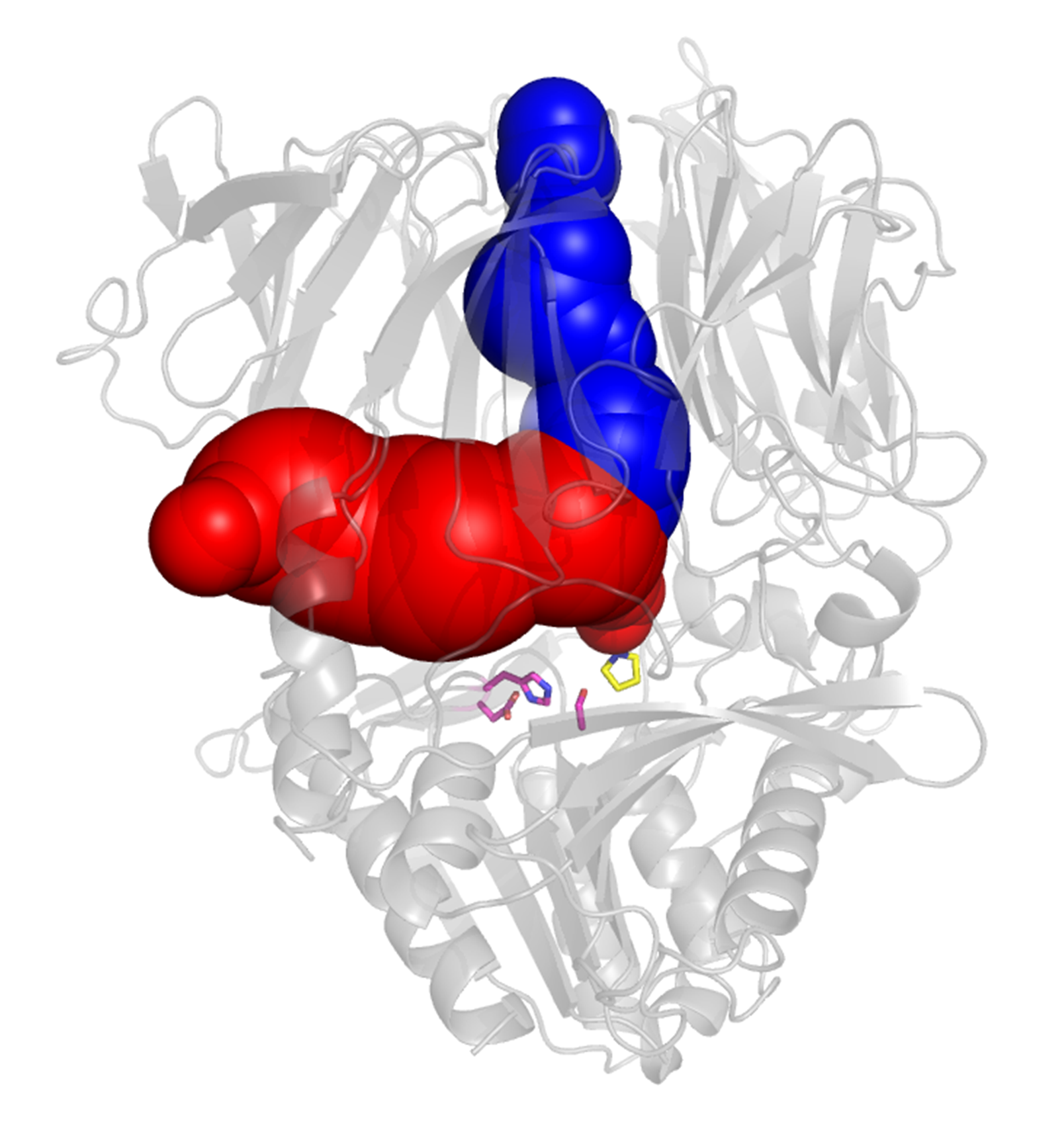

Supplement: Figure S1 — The channel system in DPP4. The main channel is represented in red, and is assumed to be the route for the substrates to access the active site. The products are released through the alternative channel (blue), both channels are showed here as identified by the Caver algorithm [39]. DPP4 deposited under PDB code 1N1M was used for the channels calculation. (http://www.pymol.org/). (TIF) [file pone.0043019.s001.tif]

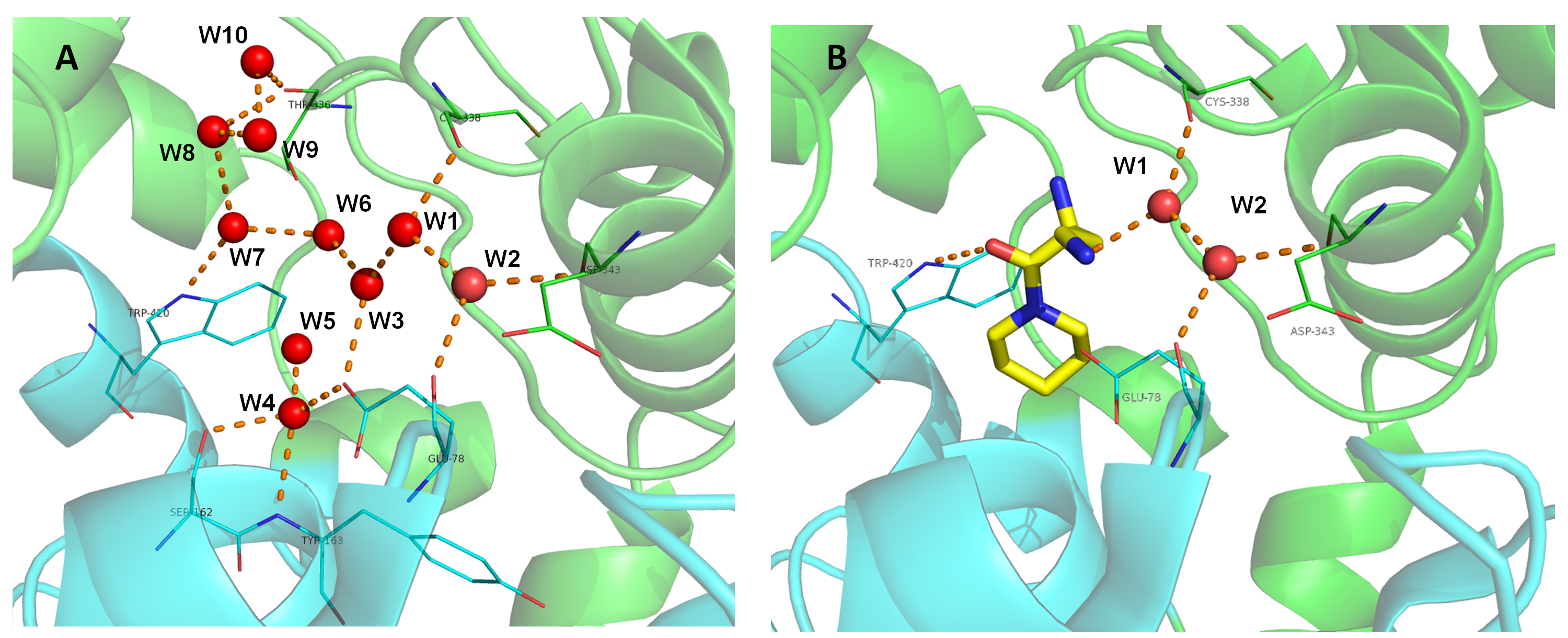

Supplement: Figure S2 — Water molecules in the active site. (A) Active site of ligand-free DPP7 with waters represented as red spheres. (B) Active site of DPP7 in complex with Dab-Pip with waters represented as red spheres and Dab-Pip as yellow sticks. Hydrogen bonds are shown as orange dashed lines. Water 1 acts as an interstitial water, bridging the interaction between the nitrogen at position 4 in the ligand with the carbonyl backbone of Cys338. The interacting residues are shown as lines. The figure was prepared using the program PyMOL (http://www.pymol.org/). (TIF) [file pone.0043019.s002.tif]

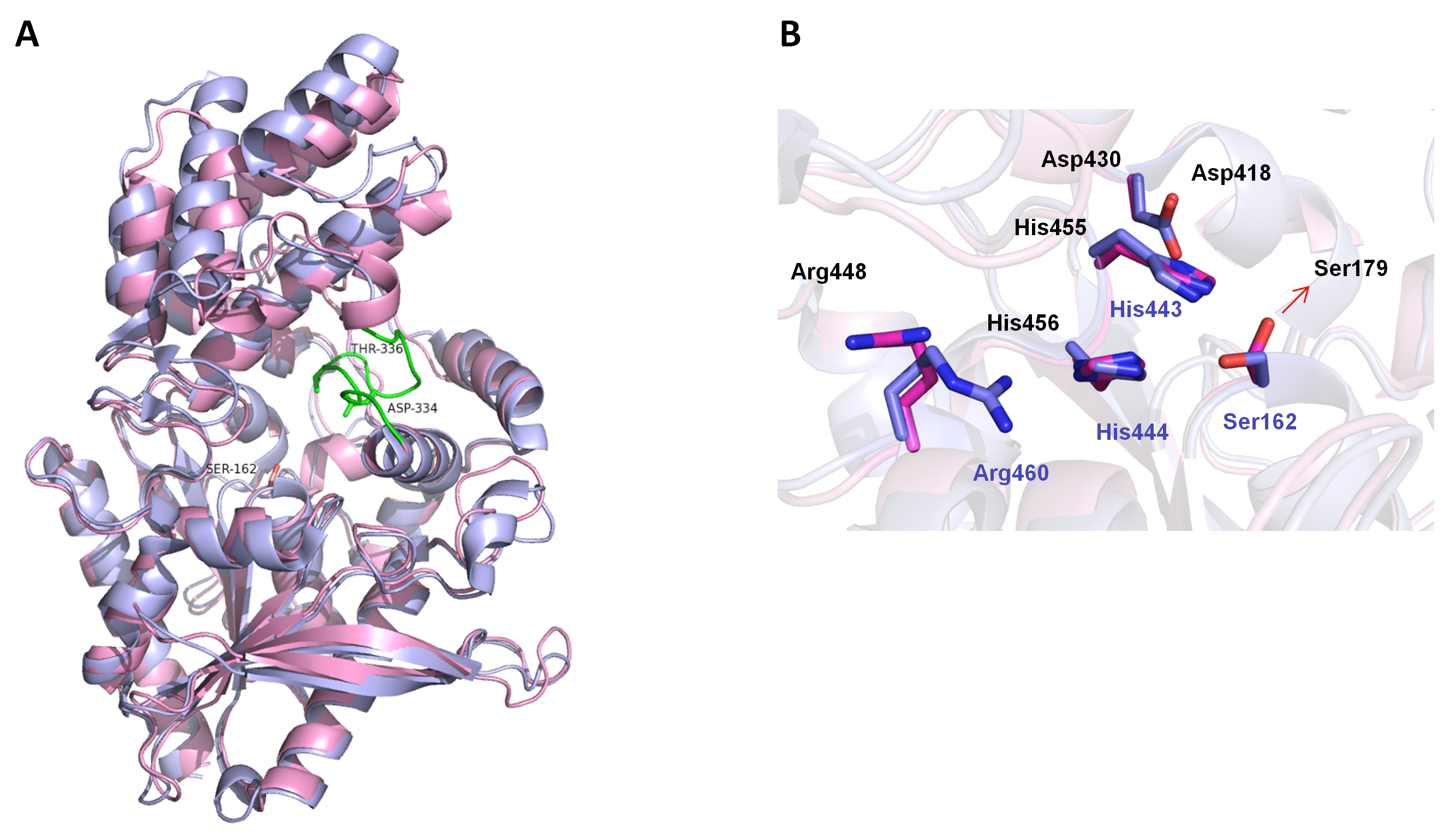

Supplement: Figure S3 — Superposition of DPP7 and PRCP. (A) Superposition of DPP7 (pink) and PRCP (blue), the insertion Trp329-Gly341 is shown as green sticks, while the catalytic Ser162 is depicted as a pink stick. (B) Zoom in the superposed active site of DPP7 (shown in pink) and PRCP (shown in blue). The corresponding amino acid numbers are shown in black. (TIF) [file pone.0043019.s003.tif]
