# Supplementary material for: Structures of Human DPP7 Reveal the Molecular Basis of Specific Inhibition and the Architectural Diversity of Proline-Specific Peptidases
Source: PLoS One. 2012 Aug 29;7(8):e43019. doi: 10.1371/journal.pone.0043019 (PMC3430648; doi:10.1371/journal.pone.0043019)
Supplement: Table S1 — Accessible surface area (in Å2) for the residues constituting the catalytic triad. (DOCX) [file pone.0043019.s004.docx]

**Table S1:** Accessible surface area (in Å^2^) for the residues constituting the catalytic triad.

|  | **ligand-free DPP7 @ 2.2 Å** | | | | **ligand-free**  **DPP7 @ 2.0 Å** | | **complex** | | | |
| --- | --- | --- | --- | --- | --- | --- | --- | --- | --- | --- |
| chain | A | B | C | D | A | B | A | B | C | D |
| Ser162 | 17.6 | 18.1 | 18.0 | 17.8 | 15.7 | 17.5 | 6.1 | 6.1 | 5.3 | 7.6 |
| Asp418 | 3.0 | 2.9 | 3.4 | 3.5 | 2.0 | 0.9 | 5.4 | 4.3 | 3.6 | 3.8 |
| His443 | 2.6 | 2.3 | 2.9 | 4.1 | 4.8 | 5.3 | 3.1 | 3.6 | 2.9 | 2.9 |
