# Supplementary material for: Structures of Human DPP7 Reveal the Molecular Basis of Specific Inhibition and the Architectural Diversity of Proline-Specific Peptidases
Source: PLoS One. 2012 Aug 29;7(8):e43019. doi: 10.1371/journal.pone.0043019 (PMC3430648; doi:10.1371/journal.pone.0043019)
Supplement: Table S2 — H-bonding distances between Ser-His and His-Asp of the catalytic triad. (DOCX) [file pone.0043019.s005.docx]

**Table S2:** H-bonding distances between Ser-His and His-Asp of the catalytic triad.

| Interactions within the catalytic triad | | distance |
| --- | --- | --- |
| **ligand-free DPP7 @ 2.0 Å** | |  |
| Chain A | |  |
| Ser162 - OG | His443 - NE2 | 2.7 Å |
| His443 - ND1 | Asp418 – OD2 | 2.7 Å |
| Chain B | |  |
| Ser162 - OG | His443 - NE2 | 2.8 Å |
| His443 - ND1 | Asp418 – OD2 | 2.6 Å |
| Chain C | |  |
| Ser162 - OG | His443 - NE2 | 2.8 Å |
| His443 - ND1 | Asp418 – OD2 | 2.7 Å |
| Chain D | |  |
| Ser162 - OG | His443 - NE2 | 2.7 Å |
| His443 - ND1 | Asp418 – OD2 | 2.6 Å |
| **ligand-free DPP7 @ 2.2 Å** | |  |
| Chain A | |  |
| Ser162 - OG | His443 - NE2 | 2.8 Å |
| His443 - ND1 | Asp418 – OD2 | 2.7 Å |
| Chain B | |  |
| Ser162 - OG | His443 - NE2 | 2.9 Å |
| His443 - ND1 | Asp418 – OD2 | 2.6 Å |
| **complex** | |  |
| Chain A | |  |
| Ser162 - OG | His443 - NE2 | 2.8 Å |
| His443 - ND1 | Asp418 – OD2 | 2.8 Å |
| Chain B | |  |
| Ser162 - OG | His443 - NE2 | 2.7 Å |
| His443 - ND1 | Asp418 – OD2 | 2.8 Å |
| Chain C | |  |
| Ser162 - OG | His443 - NE2 | 2.8 Å |
| His443 - ND1 | Asp418 – OD2 | 2.9 Å |
| Chain D | |  |
| Ser162 - OG | His443 - NE2 | 2.5 Å |
| His443 - ND1 | Asp418 – OD1 | 2.8 Å |
